# High‐Precision Printing of Complex Glass Imaging Optics with Precondensed Liquid Silica Resin

**DOI:** 10.1002/advs.202105595

**Published:** 2022-04-25

**Authors:** Zhihan Hong, Piaoran Ye, Douglas A. Loy, Rongguang Liang

**Affiliations:** ^1^ James C. Wyant College of Optical Sciences The University of Arizona 1630 E University Blvd Tucson AZ 85721 USA; ^2^ Department of Chemistry & Biochemistry The University of Arizona 1306 E. University Blvd Tucson AZ 85721‐0041 USA

**Keywords:** 3D printing, glass, micro‐optics, silica precursor, two‐photon polymerization

## Abstract

3D printing of optics has gained significant attention in optical industry, but most of the research has been focused on organic polymers. In spite of recent progress in 3D printing glass, 3D printing of precision glass optics for imaging applications still faces challenges from shrinkage during printing and thermal processing, and from inadequate surface shape and quality to meet the requirements for imaging applications. This paper reports a new liquid silica resin (LSR) with higher curing speed, better mechanical properties, lower sintering temperature, and reduced shrinkage, as well as the printing process for high‐precision glass optics for imaging applications. It is demonstrated that the proposed material and printing process can print almost all types of optical surfaces, including flat, spherical, aspherical, freeform, and discontinuous surfaces, with accurate surface shape and high surface quality for imaging applications. It is also demonstrated that the proposed method can print complex optical systems with multiple optical elements, completely removing the time‐consuming and error‐prone alignment process. Most importantly, the proposed printing method is able to print optical systems with active moving elements, significantly improving system flexibility and functionality. The printing method will enable the much‐needed transformational manufacturing of complex freeform glass optics that are currently inaccessible with conventional processes.

## Graphical abstract

A new precondensed liquid silica resin and 3D printing method are optimized for printing high‐precision, complex glass optical elements with much better thermal stability, mechanical properties, chemical resistance, and imaging performance. 3D printing has unique capabilities in fabricating optical element with freeform and discontinuous shapes, complex multielement alignment‐free optical systems, and optical systems with moving element.

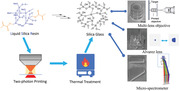

## 1 Introduction

Inorganic glass has been used for fabricating optics for hundreds of years. Although more and more optics are fabricated from organic polymers due to its light weight and low‐cost, inorganic glass still has an irreplaceable position in the optical imaging because of its much better thermal stability, mechanical properties, chemical resistance, and imaging performance in ultraviolet (UV), near‐infrared (NIR), and infrared (IR) regions.^[^ ^1^, ^2^ ^]^

Glass micro‐optics have been widely used in consumer products, medical devices, sensors, optical communications, and etc.^[^ ^3^, ^4^ ^]^ Traditional grinding/polishing, commonly used to fabricate glass optics, is not efficient in fabricating micro‐optics and is not capable of fabricating freeform micro‐optics with discontinuous surfaces. Precision press glass molding, developed for mass production of glass optics, is a preferred method for fabricating low‐cost micro‐optics, but cannot be used to fabricate multielement components and freeform optics with microstructures.^[^ ^5^ ^]^ In recent years, additive manufacturing (AM), or 3D printing, has been used to fabricate small and complicated structures that conventional techniques cannot achieve. A variety of AM techniques have been investigated to print glass objects using different materials.^[^ ^6^, ^7^, ^8^, ^9^, ^10^, ^11^, ^12^, ^13^, ^14^, ^15^, ^16^, ^17^ ^]^ However, considering the strict requirements on surface shape and quality for optical applications, the relatively low resolution of some AM techniques (e.g., fused filament fabrication, direct ink writing, and stereolithography) limits their printing glass optics unless post‐process (e.g., polishing) is applied to the printed parts, which is not ideal and sometimes impossible. AM technique based on two‐photon polymerization (TPP) becomes the best candidate to print glass micro‐optics since it has much higher printing resolution.^[^ ^15^, ^16^, ^17^ ^]^

Until recently, TPP‐based AM technique has been used to print high resolution, microsized optics based on organic polymers.^[^ ^17^, ^18^, ^19^, ^20^ ^]^ Micro‐optics were 3D printed from silica particles in an organic resin by Kotz et al. using a TPP direct laser writing (DLW) method.^[^ ^11^ ^]^ Thermal degradation and sintering at 1300 °C afforded silica glass with a linear shrinkage of ≈26%. Microlenses with diameters of hundreds of micrometers were obtained with *R*_a_ ≈ 6 nm and *S*_a_ of hundreds of nanometers. We reported a TPP printing method with a solvent‐free, pre‐condensed liquid silica resin (LSR) for fabricating microglass optics with relatively simple structures (e.g., single lens or grating).^[^ ^15^ ^]^ Transparent glass optics can be obtained after thermal treatment at 600 °C in the air with linear shrinkage as low as 17%. The *S*_a_ and *S*_q_ of printed semisphere lenses reached 4.3 and 5.6 nm, respectively. The solvent‐free LSR used in our previous work was synthesized based on acid‐catalyzed polymerization of tetramethoxysilane (TMOS) together with substoichiometric amount of water (water solution) and 6.5 mol% of methacryloxymethyltrimethoxysilane (MMTS) as photocurable moiety. Recently, we observed a limitation that the deformation may happen during the printing and thermal treatment processes if a structure with high aspect ratio is printed, mainly due to the relatively low number of crosslinked points in the printed structure. When 3 lens objective with high aspect ratio (diameter of 50 µm and height of 100 µm) was printed, the supporting structure was not strong enough to support the whole objective after printing.

In this work, we report our recent progress in printing imaging micro‐optics with newly optimized LSR. The printed structure can be converted to transparent silica glass at a temperature as low as 600 °C. Microsized single, freeform, multicomponent glass optics with well‐controlled profile accuracy (RMS surface roughness ≤ 5 nm) have been fabricated. The performances of all optical systems have been evaluated to demonstrate the potentials for practical applications.

## 2 Precondensed Liquid Silica Resin with Better Curing and Mechanical Properties for Optical Applications

**Figure 1:**
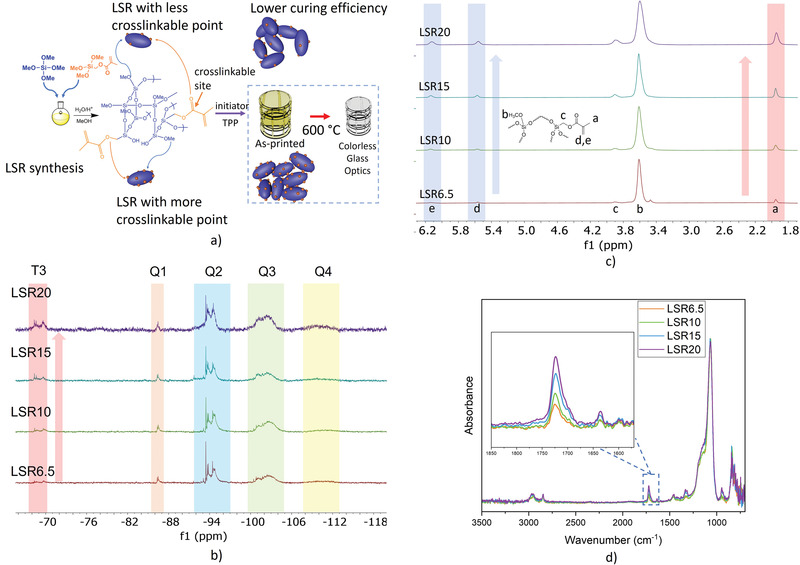
a) Scheme of LSR synthesis and fabrication of glass micro‐optics. The LSR sol can be considered as soft, flexible, and nanometer‐sized particles. b) The ^29^Si NMR spectra of LSRs. c) The^1^H NMR spectra of LSRs. d) The FTIR spectra of LSRs. After normalizing all curves based on Si‐O peak (1066 cm^–1^), the ratio of integrations of C═O (1723 cm^–1^) peaks from LSR6.5 to LSR10, to LSR15, to LSR20 is 1:1.5:2.3:3.0.

To address the deformation challenge, we synthesized a series of LSRs with increased crosslinkable points (**Figure** **1**a), by adjusting the ratio of MMTS during synthesis. LSRs with different ratios of MMTS varying from 6.5 mol% to 20 mol% were prepared (LSR6.5, LSR10, LSR15, LSR20). Si NMR was used to characterize the chemical structures of LSRs. Peaks in Q_1_ (‐86 to ‐87 ppm), Q_2_ (‐93 to ‐96 ppm), Q_3_ (‐100 to ‐105 ppm), and Q_4_ (‐107 to ‐115 ppm) regions were observed in all LSRs’ spectra with almost the same integration ratio (*Q*_2_ ≈ *Q*_3_ >> *Q*_4_ > *Q*_1_), indicating that the hydrolysis and condensation during synthesis could be well controlled by strictly controlling the ratio of H_2_O:Si (Figure 1b). These results are consistent with the spectrum of liquid silica prepared from pure TMOS.^[^ ^21^ ^]^ Since MMTS was also introduced as comonomer, T_3_ (‐68 to ‐70 ppm) peaks were also observed. The multipeaks in T_3_, Q_2_, and Q_3_ regions indicate that the LSRs are a mixture of linear, cyclic, and cage‐shaped species. From LSR6.5 to LSR20, the increased intensity of T_3_ peaks is consistent with the increased ratio of methacrylate group in the system, which is also confirmed by the increased intensity of methacrylate peaks (6.13, 5.97, 3.90, and 1.95 ppm) in ^1^H NMR (Figure 1c) and the increased intensity of C═O peak (1723 cm^–1^) in normalized FTIR spectrum (normalized by the Si—O peak at 1066 cm^–1^) (Figure 1d).

**Figure 2:**
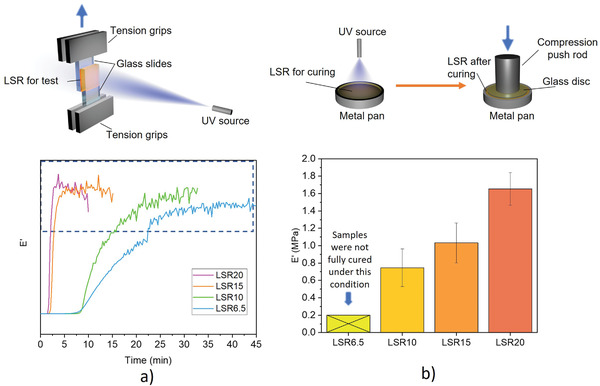
a) The DMA (tension) results of LSRs between two glass slides exposed to UV. The zig‐zag part of each curve in the dashed region refers to the crack formation and broken of LSR at the end of the test, which could be caused by the heat during UV exposure. The gel time of each curve was determined by crossing the baseline with the tangent line of point on curve that *E*’ has the highest raising rate. b) The *E*’ of LSRs after curing by normal UV. The tests were conducted under compression mode. The LSR6.5 cannot be fully cured under the condition used for UV curing test.

When the percentage of MMTS is increased, shrinkage increases as well during thermal treatment, which is caused by eliminating a greater volume of the organic methacrylate group.^[^ ^15^ ^]^ To achieve the high‐quality printing for optics, the shrinkage should be kept as low as possible for better control in shape and surface quality. Therefore, it is important to seek an optimized ratio of MMTS that provides sufficient crosslinking points with minimized shrinkage during pyrolysis. The gel point time of LSRs under UV irradiation was measured using dynamic mechanical analysis (DMA) under a tension mode. The LSRs were placed between two glass slides. The UV light was applied to the LSR after a 2.5 min stable measurement without UV irradiation. The results indicated that LSR10 had a similar curing efficiency as the LSR6.5. When the ratio of MMTS was increased to 15 mol%, a substantial decrease of gel point time (from 8.5 to 2 min) under the same curing conditions was observed (**Figure** **2**a), indicating a much higher curing efficiency. Further increasing the MMTS to 20 mol% did not improve the curing efficiency much. The storage modulus (*E*′) of UV‐cured LSR was also measured by DMA to evaluate the mechanical properties after curing. To minimize the heat generated by UV, limited UV power (≈10 W cm^−1^,^[^ ^2^ ^]^ the UV source was 5 cm away from the sample) and 5 min exposure time was used. Under such conditions, the LSR6.5 could not be fully cured, and the DMA results were not stable. Meanwhile, LSRs with increased MMTS ratio from 10 mol% to 20 mol% show the increased *E*’ from 0.74 to 1.65 MPa (Figure 2b). Although the LSR20 had better mechanical properties than LSR15 after curing, to minimize the shrinkage we selected LSR15 to investigate the printing process for precision optics for imaging applications. The linear shrinkage of LSR15 at 600°C was measured as 22% (Figure S1, Supporting Information), which is higher than the shrinkage of LSR6.5 (17%). This is because the LSR15 contains more organic parts compared to LSR6.5.^[^ ^15^ ^]^

## 3 High‐Precision Printing of Glass Optics with the Optimized Precondensed LSR

A custom printing system was built to study the printing process. A 780 nm femtosecond fiber laser source with ≈150 fs, 77 MHz, and maximum power of 130 mw was used for two‐photon polymerization.^[^ ^15^ ^]^ The collimated laser beam was expanded to 5.5 mm diameter by a 5× beam expander. The full‐width half maximum (FWHM) of the beam was 3.24 mm, 65% filling the objective aperture of the oil immersion objective (NA = 1.25). The optical elements were printed with a 1.52 nJ pulse energy and a printing speed of 2.5 mm s^−1^.

The printed elements were immersed in propylene glycol monomethyl ether acetate (PGMEA) for 5 min after printing and then immersed in alcohol for another 5 minutes. After washing away the uncured material, the elements were kept at room temperature before thermal treatment. The multistage thermal treatment was finished in a Vulcan 3‐550 furnace. The printed elements were first heated to 200 °C (1 °C min^−1^) and held for 3 h, followed by being heated to 350 °C (0.5 °C min^−1^) and held for 3 h, and then by a heating ramp to 600 °C (0.5 °C min^−1^). After that, the elements were held for another 3 h before they were cooled to room temperature slowly.

**Figure 3:**
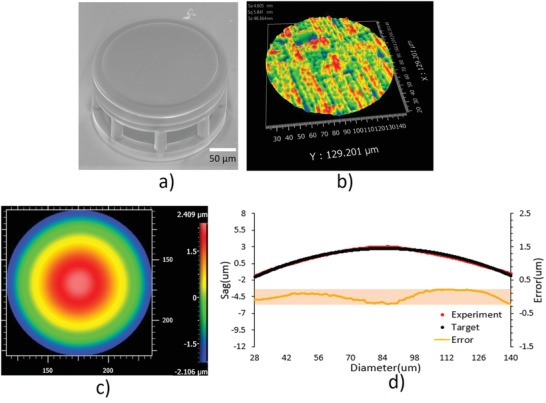
Surface roughness and shape of the printed optical surface. a) SEM image and b) surface roughness of a printed flat element. c) The measured surface shape of a printed spherical surface with a radius of 370 µm and d) the deviation of the printed surface profile from the designed shape. The measurement was performed with Zygo Newview 8300 optical profilometer.

To demonstrate the printing capabilities and evaluate the printing quality for optical imaging applications, we first printed a flat element as shown in **Figure** **3**a, the flat element was printed on an array of supporting pillars.^[^ ^15^ ^]^ Figure 3b shows that the surface roughness is better than 6 nm, which could be further improved by optimizing the printing system with better motion‐controlled mechanisms. Figure 3c is the measured surface shape of a printed spherical surface with a radius of 370 µm and Figure 3d shows the deviation of the surface profile from the designed radius, demonstrating that the printed lens has a P–V better than 450 nm and an RMS better than 135 nm. No surface deformation was observed before and after thermal treatment. This is attributed to the precondensed resin. The resin prepared by sol‐gel chemistry is highly homogenous, which avoids the uncontrolled deformation during shrinking. Moreover, the optimized equal‐arc printing method^[^ ^15^ ^]^ ensures the high printing accuracy to for the industry standards.

**Figure 4:**
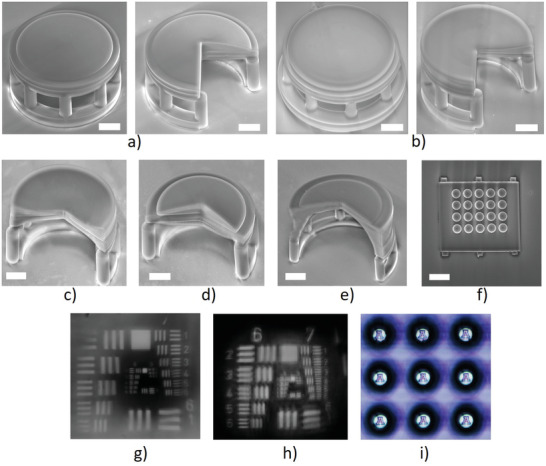
SEM images of a) plano‐convex singlet, b) plano‐concave singlet, c) bi‐concave singlet, d) bi‐convex singlet, e) meniscus lens, and f) lenslet array. g,h) Images of 1951 USAF resolution target captured by the plano‐convex singlet in (a) and the plano‐concave singlet in (b). i) The image of University of Arizona logo captured by the lenslet array in (f). Scale bar: 50 µm.

**Figure** **4** shows SEM images of singlets with common shapes. Figure 4a is the plano‐convex lens, the left image is the entire lens, and the right image is the ¾ sectioned lens to show the lens shape. The layer‐like structure in the cross‐section image was caused by the printing process; the internal property is homogenous. Figure 4b–f is SEM images of planoconcave, biconcave, biconvex, meniscus lens, and lenslet array. This study demonstrates that the reported printing material and process is able to print the lenses with common shapes. Figure 4g–i is the images formed by the printed lenses. The printed lens first imaged the target to an intermediate image which was then relayed to the CMOS camera by a microscope, as laid out in Figure 7c. Figure 4g,h is the images of the 1951 USAF resolution target captured by the plano‐convex singlet in Figure 4a and the planoconcave singlet in Figure 4b. The image quality was degraded because the singlet didn't have sufficient freedoms to correct aberrations. The contrast, particularly in Figure 4g, was relatively low due to the stray light. Figure 4i is the image of the University of Arizona logo captured by the lenslet array in Figure 4f.

**Figure 5:**
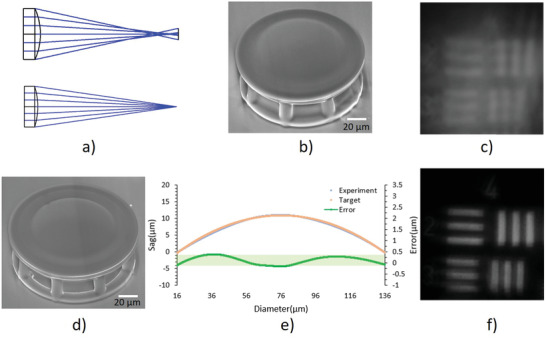
Capability in printing aspherical surface. a) Ray tracing of the plano‐convex singlet with the spherical surface (top) and aspherical surface (bottom), b) SEM image of the plano‐convex single with spherical surface, c) image of the element of the fourth group in USAF resolution target captured by the lens in (b), d) SEM image of the plano‐convex single with aspherical surface, e) surface deviation of the printed and designed aspherical surface, and f) image of the element of the fourth group in USAF resolution target captured by the lens in (d).

One of the key advantages of the reported printing material and process is its capability in printing aspherical and freeform elements without additional processes. **Figure** **5**a plots the ray tracing diagrams of two plano‐convex singlets with the spherical surface (top) and aspherical surface (bottom) with the same base radius of 220 µm, showing the aspherical surface is very effective in minimizing spherical aberration. The aspherical surface was an even aspherical surface described by the following equation:

(1) $$Z\left(r\right)=\frac{cr^{2}}{\;1+\sqrt{1-\left(1+\kappa \right)c^{2}r^{2}}\;}+\alpha_{2}r^{2}+\alpha_{4}r^{4}+\alpha_{6}r^{6}$$

where *R*  =  220 µm, *κ*  =   − 1, *α*_2_ =  0.474, *α*_4_ =  6.4036, and *α*_6_ =   − 41.454.

To compare the imaging performance, both lenses were printed. Figure 5b–d is the SEM images of the planoconvex spherical singlet and aspherical singlet. Figure 5e compares the surface profiles of the designed and printed aspherical surfaces, the peak‐valley surface deviation is less than 1 wave, and RMS surface deviation is less than ¼ wave, demonstrating the proposed 3D printing process is able to print aspherical surface precisely. Figure 5c–f is the images of the elements of the fourth group in the 1951 USAF resolution target, clearly validating that the aspherical surface is effective in reducing the spherical aberration for better image quality. The reason that the resolving powers of the lenses in Figure 5 were lower than that of the lenses in Figure 4 was that the numerical apertures of the lenses in Figure 5 were smaller than that of the lenses used in Figure 4.

**Figure 6:**
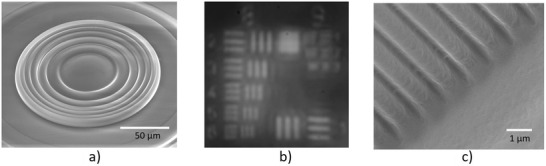
3D printed optical elements with discontinuous structures. a) SEM image of a Fresnel surface, b) the image of 1951 USAF resolution target, and c) SEM image of a grating with a period of 1.1 µm.

It is well recognized that traditional grinding and polishing methods are incapable of fabricating glass optical elements with structures. However, the proposed 3D printing technology is able to print discontinuous optical components (**Figure** **6**), further enriching the feasibility of the glass imaging optics integration. Figure 6a is the SEM image of a Fresnel surface, and Figure 6b is the image of the 1951 USAF resolution target formed by this printed Fresnel lens. It was able to resolve the element 6 in group 8; as expected, the contrast was reduced due to the stray light from the Fresnel structures. Figure 6c is one more example which is the SEM image of a grating with a period of 1.1 µm. This unique capability will significantly reduce the fabrication cost of prototyping discontinuous optical surfaces, potentially enabling more unique applications.

## 4 High‐Precision Printing of Complex Imaging Systems

**Figure 7:**
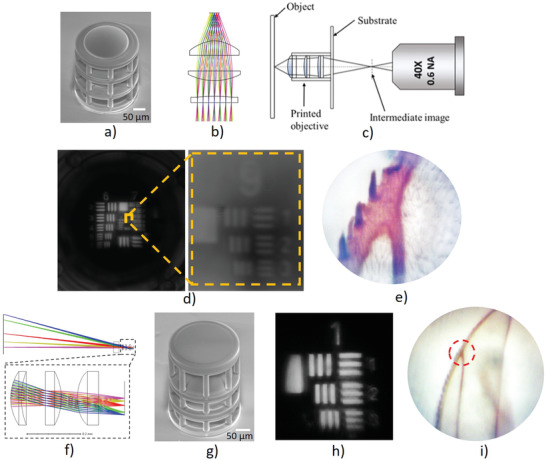
3D printed micro‐objective with three elements. a) SEM image of the printed micro‐objective, b) optical layout, c) setup for evaluating the imaging performance of the printed micro‐objective, b) the image of 1951 USAF resolution target, and e) image of a housefly wing captured by the printed micro‐objective. f) Optical layout of endoscope objective with three elements. g) SEM image of the printed micro‐objective. h) The image of 1951 USAF resolution target, and i) image of a housefly wing captured by the printed endoscope objective.

A very time‐consuming and error‐prone process in building complex optical imaging systems is assembling and aligning optical elements, particularly for micro‐optical systems without sufficient freedoms for alignment. One of the key advantages of 3D printing is that all elements can be fabricated together without further alignment, significantly simplifying the process of developing new optical systems. **Figure** **7**a is the SEM image of a three‐element micro‐objective with the optical layout in Figure 7b. All three elements were printed as a single objective, the spaces between elements, as well as the tilt and decenter of each element, were controlled precisely by the computer‐control motion stage. To test its imaging performance, the image of the USAF 1951 resolution target formed by the micro‐objective was relayed to the CMOS sensor by a microscope with a 0.5NA microscope objective (Figure 7c). As shown in Figure 7d, the printed micro‐objective was able to clearly resolve Element 3 in Group 9, corresponding to the resolution of 780 nm. Figure 7e shows images of the house fly wing, further demonstrating the imaging performance of the printed glass micro‐objective.

In contrast to high resolution, small field of view objective, an endoscope objective with large field of view and lower solution was also fabricated and evaluated. Figure 7f,g is the optical layout and the SEM image of the printed endoscope objective. Figure 7h is the image of the elements in the first group in 1951 USAF resolution target. Figure 7i is the image of the same house fly wing, showing the larger field of view and lower resolution than that of the micro‐objective in Figure 7a. The red dot circle in Figure 7i shows the region imaged by the micro‐objective and displayed in Figure 7e.

**Figure 8:**
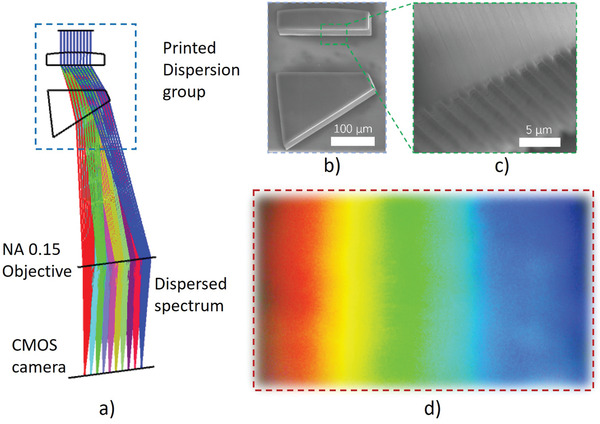
Microspectrometer with printed dispersion assembly. a) Optical layout, b) SEM image of the printed dispersion assembly, c) SEM image of the lens surface with grating, and d) dispersed spectrum captured by the CMOS camera.

As demonstrated in Figure 6, the proposed printing technique is able to print optical elements with microstructures. This capability enables unique applications not possible with the traditional fabrication methods. **Figure** **8** demonstrates a compact spectral imaging system. Figure 8a is the layout of the micro‐spectrometer with the printed dispersion assembly, which consists of a lens with grating in the flat surface and a dispersion prism. Figure 8b is the SEM image of the printed dispersion assembly with the lens and prism printed on the same substrate, and Figure 8c is the SEM image of the lens flat surface with grating. The collimated light from a white LED illuminated the dispersion group and then focused to the CMOs camera by a microscope objective. The dispersed spectrum distribution was clearly recorded in the CMOS camera as shown in Figure 8d. This example demonstrates that more complex, alignment‐free optical systems with different types of optical elements can also be printed.

**Figure 9:**
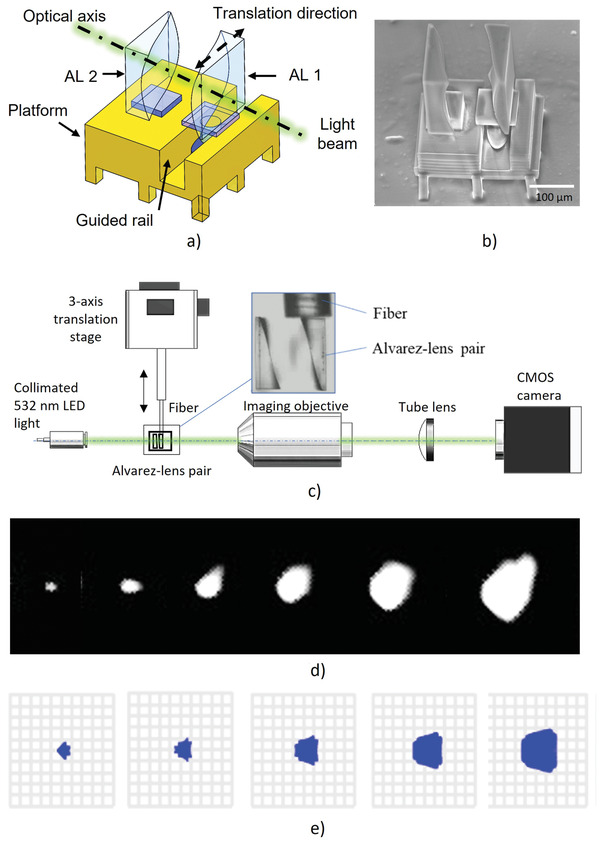
Alvarez lens with the movable element. a) Schematic diagram and b) SEM image of the assembled Alvarez lens. Experiment to demonstrate the performance of the printed Alvarez lens: c) Experimental setup, d) measured spot sizes, and e) simulated spot sizes. (Videos S1 and S2, Supporting Information).

To further demonstrate the unique capability of the proposed printing technique in printing freeform surface, we printed an Alvarez lens pair, one of which was movable.^[^ ^22^ ^]^ **Figure** **9**a is the schematic diagram, a platform with a guided rail was printed first, and then the first lens was printed onto the platform directly. The second lens was printed separately and then mounted to the guided rail. The Alvarez lens was defined by the following equations:

(2a) $$Z_{1}\left(X,Y\right)=A\left(\frac{1}{3}X^{3}+XY^{2}\right)+C$$

(2b) $$Z_{2}\left(X,Y\right)=-A\left(\frac{1}{3}X^{3}+XY^{2}\right)+C$$

where *A* = 0.0216 and *C* = 0.036. The distance between two lenses was 110 µm. This Alvarez lens focused the collimated beam to different locations as one of the lenses moved.

Figure 9b is the SEM image of the assembled Alvarez lens. A testing system was built to evaluate the function of the printed Alvarez lens as shown in Figure 9c. A collimated 532 nm LED light illuminated the assembled Alvarez lens. A small fiber was mounted to a 3‐axis translation stage to move one of the Alvarez lenses as shown in the inset. A microscope was used to image the plane where the initially focused spot was located to the CMOS camera. During the movement of one Alvarez lens, the microscope was not moved to follow the focal point to show the change in beam size. As shown in Figure 9d, the spot size on the camera increased as the Alvarez lens was moved. Figure 9e is the simulated measured spot size (not the focal point) at the image plane. Compared Figure 9d,e, the through‐movement change of the spot size matched well, with some minor differences in shape, mainly due to the beam shape of the input light. Optical systems with movable elements, such as zoom lens, are often designed and fabricated to meet some special needs. For this type of optical systems, the challenge is the moving element. This study has demonstrated the great potentials of the reported 3D printing method and materials in future various applications.

## 5 Conclusion

We have developed and characterized the optimized precondensed liquid silica resin with higher curing speed, better mechanical properties, lower thermal treatment temperature without sintering, reduced shrinkage, and good optical performance. We have also demonstrated the precision TPP 3D printing process for complex glass optical systems and evaluated their performance for imaging applications. Compared to the 3D printed polymer optics, the glass optics has much better thermal stability, mechanical properties, chemical resistance, and imaging performance in UV, NIR, and IR regions. Compared to the traditional polishing and molding methods, 3D printing has unique capabilities in fabricating optical element with freeform and discontinuous shapes, complex multielement alignment‐free optical systems, and optical systems with moving element. Based on the measured surface quality and shape deviation, as well as the image quality, we believe that 3D printing of glass imaging optics will play a significant role in precision optical imaging very soon.

## Conflict of Interest

R.L. is the founder of Light Research Inc., which didn’t support this research.

## Supporting information

Supporting Information

Supplemental Video 1

Supplemental Video 2

## Data Availability

The data that support the findings of this study are available in the supplementary material of this article.
