# Supplementary material for: High‐Precision Printing of Complex Glass Imaging Optics with Precondensed Liquid Silica Resin
Source: Adv Sci (Weinh). 2022 Apr 25;9(18):2105595. doi: 10.1002/advs.202105595 (PMC9218758; doi:10.1002/advs.202105595)
Supplement: Supplementary file 1 — Supporting Information [file ADVS-9-2105595-s001.pdf]

ADVANCED SCIENCE
Open Access

Supporting Information

for *Adv. Sci.*, DOI 10.1002/advs.202105595

High-Precision Printing of Complex Glass Imaging Optics with Precondensed Liquid Silica Resin

Zhihan Hong, Piaoran Ye, Douglas A. Loy and Rongguang Liang**

Supporting Information

High-precision printing of complex glass imaging optics with pre-condensed liquid silica resin

ZHIHAN HONG,[1,†] PIAORAN YE,[2,†], DOUGLAS A. LOY,[2,3,4] AND RONGGUANG LIANG[1,*]

[†] *These authors contributed equally to this work*
[1]*Wyant College of Optical Sciences, The University of Arizona, 1630 E University Blvd, Tucson, AZ 85721, USA*
[2]*Department of Chemistry & Biochemistry, The University of Arizona, 1306 E. University Blvd, Tucson, AZ 85721-0041, USA*
[3]*Department of Materials Science & Engineering, The University of Arizona, 1235 E. James E. Rogers Way, Tucson, AZ 85721-0012, USA*
[†]*Equal contribution*
[4]*daloy@arizona.edu*
*rliang@optics.arizona.edu

Materials and Methods

Materials

Tetramethoxysilane (TMOS, 98%) and methacryloxymethyltrimethoxysilane (MMTS) were purchased from Gelest. 4,4'-Bis(diethylamino)benzophenone (BEBP), monomethyl ether hydroquinone (MEHQ), and Propylene glycol monomethyl ether acetate (PGMEA) was purchased from Sigma-Aldrich. The methanol was refluxed with magnesium and distilled before use. All other chemicals were used as received unless a specific statement.

Synthesis of pre-condensed polysilsesquioxane (LSR) for printing

MMTS with a designed ratio (from 6.5 mol% to 20 mol% regarding of total amount of silane) were mixed with TMOS and methanol in a flame dried 50 mL round bottom flask. The concentration of MMTS and TMOS together was fixed as 0.022 mol in 8.4 g of methanol. MEHQ (10 mg, 0.08 mmol) was added as an inhibitor to prevent the polymerization of MMTS during the pre-condensation. Then, dilute HCl (1 M, 1.45 eq of H_2O to Si) was added dropwise under magnetic stirring. The solution in the flask was heated at 57 °C for 4 h. After that, the methanol and HCl were evaporated under vacuum (~ 1mmHg) for 18 h. Then, BEBP (0.8 wt% to final LSR) was dissolved in 1 mL of dry methanol followed by being mixed with the pre-condensed LSR. Upon the clear and homogeneous solution was formed, the yellow solution was transferred to a 20 mL glass vial through a syringe equipped with a 0.2 μm filter. Methanol was removed under vacuum (~ 1 mmHg) before printing. This material can be stored in a sealed vial in freezer for at least two months before usage.

Characterization

Infrared (IR) spectra were obtained with a Thermo Scientific Nicolet iS50R using a Harrick MVP-Pro™ Single Reflection ATR Microsampler. Nuclear magnetic resonance (NMR) was obtained using a Bruker DRX 500 MHz. For ^{29}Si NMR, 300 mg of LSR was dissolved in 1 mL $CDCl_3$. 15mg of chromium acetylacetonate was added as relaxation agent. Scanning Electron Microscope (SEM) images were taken using FEI Inspect Scanning Electron Microscope. All SEM images were taken under low vacuum mode without any coatings except other statement. The surface profile was measured by Zygo Newview 8300 white light interference microscope.

Measurement of curing efficiency and storage modulus of LSR

The curing efficiency and storage modulus (E') were measured using NETZSCH DMA 242E. To measure the curing efficiency, two glass slides were clamped by the sample holder (tension) with an overlapped length of 5 mm. The overlapped parts of these two slides were not physically contacted. LSR was placed to form a thin liquid layer to fill the gap between the two slides within the overlapped region. The UV light was generated by Omnicure 2000 with 20% output power. The UV was applied to the LSR after the measurement started. After the measurement, the gel time of each curve was determined by crossing the baseline with the tangent line of point on curve that E' has the highest raising rate.

To measure the E' of the cured LSR, LSRs were firstly placed in thin aluminum pans. UV was generated by Omnicure 2000 with 30% output power and exposed to each pan for 300 s to cure the LSR. After curing, a thin glass disc (3 mm diameter) was put between the LSR and the pushrod of the DMA. The measurements were down with compression mode. Since the cured LSRs were measured within the pan, the obtained data is comparable with each other but not the real E' of the cured LSRs.

Printed optical element before and after thermal treatment.

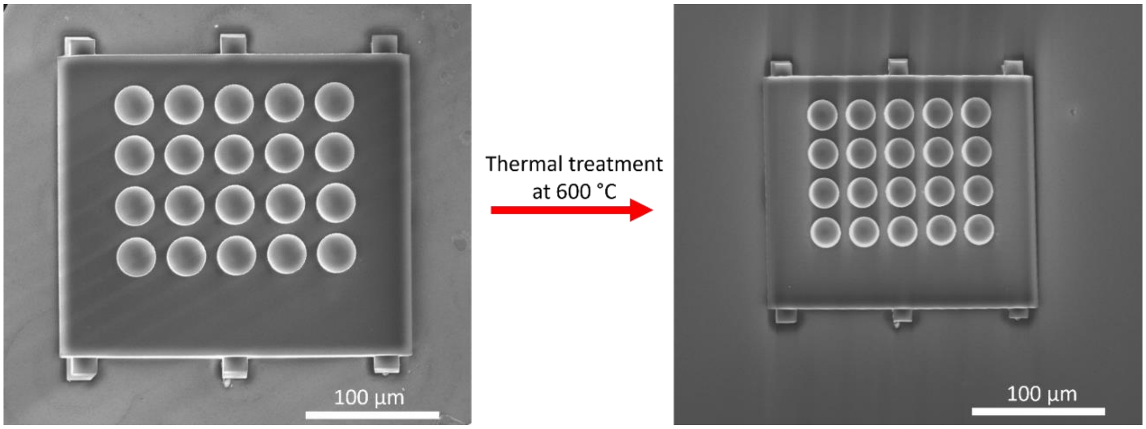

Figure S1. Lens array printed with LSR15 before and after thermal treatment at 600 °C

High-precision printing of complex glass imaging optics with pre-condensed liquid silica resin

Zhihan Hong[†], Piaoran Ye[†], Douglas A. Loy[*], and Rongguang Liang[*]

Z. Hong, Prof. R. Liang
James C. Wyant College of Optical Sciences
The University of Arizona
1630 E University Blvd
Tucson, AZ 85721, USA
Email: rliang@optics.arizona.edu

P. Ye, Prof. D. A. Loy
Department of Chemistry & Biochemistry
The University of Arizona
1306 E. University Blvd
Tucson, AZ 85721-0041, USA
Email: daloy@arizona.edu

Video1.mp4: Active tuning of the Alvarez lens pair: The second lens of the Alvarez lens pair was moved laterally by a small fiber which was driven by a computer-controlled motor.

Video2.mp4: Performance of active tuning of the Alvarez lens pair: The beam transmitted through the Alvarez lens pair during the movement of the second lens. The video was focused at the fixed plane and the field of view was larger. The Alvarez lens pair was located in the central left.
